# Calcium-mediated force–interval relationship drives post-extrasystolic potentiation in premature ventricular complexes: a computational study

**DOI:** 10.1007/s10237-026-02119-w

**Published:** 2026-08-30

**Authors:** Sjoerd Vossen, Nick van Osta, Guilherme Pedro Carvalho Lourenço, Sérgio Matoso Laranjo, Joost Lumens

**Affiliations:** 1 Department of Biomedical Engineering, Cardiovascular Research Institute Maastricht (CARIM), Maastricht University, Maastricht, The Netherlands; 2 Pediatric Cardiology Department, Hospital de Santa Marta, Unidade Local de Saúde de S. José, 1150-199 Lisbon, Portugal; 3 Comprehensive Health Research Center, NOVA Medical School, NMS, Faculdade de Ciências Médicas, FCM, Universidade NOVA de Lisboa, 1169-056 Lisbon, Portugal; 4 Medtech, AI, RWE and Digital Health Knowledge Center, NOVA Medical School, NMS, Faculdade de Ciências Médicas, FCM, Universidade NOVA de Lisboa, 1169-056 Lisbon, Portugal; 5 Digital Data Design Institute at Nova SBE and NOVA Medical School (D^3 Institute), Campus de Carcavelos, Rua da Holanda, 1, 2775-405 Carcavelos, Portugal

**Keywords:** Computational modeling, Force–interval relationship, Heart, Hemodynamics, Post-extrasystolic potentiation, CircAdapt

## Abstract

Premature ventricular complexes (PVCs) are common cardiac arrhythmias that can lead to cardiomyopathy when frequent. Post-extrasystolic potentiation (PESP), which is the transient increase in contractility following a PVC, may serve as a predictive marker for heart failure risk; yet, the underlying calcium-mediated mechanisms and their relative contribution compared to loading conditions remain poorly understood. We integrated a mechanochemical model coupling intracellular calcium dynamics to sarcomere mechanics within the CircAdapt closed-loop cardiovascular framework. A novel calcium source model incorporating the force–interval relationship was calibrated using experimental canine data. We simulated single PVCs across varying coupling intervals and systematically investigated the contributions of calcium dynamics versus loading conditions to PESP, quantified as changes in systolic blood pressure (∆SBP), maximum rate of left ventricular pressure rise (∆max(d*P*_Lv_/d*t*)), and left ventricular ejection fraction (∆LVEF). The calcium-based force–interval relationship reproduced experimental mechanical restitution curves with high accuracy (RMSE 9.59 ± 0.08%). Shorter coupling intervals reduced premature beat contractility while enhancing PESP in subsequent beats. Systematic variation of preload, afterload, and intrinsic contractility revealed that calcium dynamics reproduce the observed PESP patterns, with loading conditions as modulators. Notably, ∆max(d*P*_Lv_/d*t*) and ∆SBP responded differently, with ∆SBP exhibiting complex non-monotonic behavior. The model qualitatively reproduced pressure–volume patterns from a single clinical quadrigeminy case. This study demonstrates that a calcium-based force–interval formulation reproduces the qualitative features of PESP within this framework, with preload and afterload modulating the pattern of beat-to-beat pressure response. The divergent behavior among contractility metrics emphasizes the need for multimetric assessment. This framework enables distinguishing intrinsic myocardial dysfunction from extrinsic loading effects, facilitating patient-specific risk stratification in PVC-induced cardiomyopathy.

**Supplementary Information:**

The online version contains supplementary material available at https://doi.org/10.1007/s10237-026-02119-w.

## Introduction

Premature ventricular complexes (PVCs) are among the most common cardiac arrhythmias, yet their clinical consequences remain difficult to predict. Although PVC burdens of roughly 10–20% are associated with increased cardiomyopathy risk, individual susceptibility varies substantially: Some patients retain normal function despite burdens approaching 50%, while others exhibit marked dysfunction at burdens as low as 10% (Baman et al. 2010; Latchamsetty and Bogun 2019; Marcus 2020; de Lavallaz et al. 2022). This broad variability in vulnerability to PVC-induced cardiomyopathy complicates individualized risk stratification and the timing of therapeutic intervention (Dukes et al. 2015).

Post-extrasystolic potentiation (PESP), the transient increase in contractility following a premature beat, has been proposed as a potential biomarker for identifying at-risk patients, with clinical studies showing associations with mortality and animal models suggesting predictive value for PVC-induced cardiomyopathy development (Sinnecker et al. 2014; Kowlgi et al. 2020). However, clinical implementation of PESP assessment is limited by uncertainties regarding: (1) the mechanistic basis for divergent responses among contractility metrics and (2) the relative contributions of intrinsic calcium dynamics versus extrinsic loading conditions to PESP magnitude (Cooper 2018; Kowlgi et al. 2020).

While PESP results from altered calcium cycling during and after premature beats (Bers 2002; Sprenkeler and Vos 2016), the relationship between these cellular mechanisms and clinically observable hemodynamic consequences involves complex interactions across multiple scales, from calcium dynamics at the sarcomere level through tissue mechanics to whole-organ pump function and systemic circulation. Computational modeling offers a systematic framework to investigate these multiscale interactions, allowing translation of cellular perturbations to patient-level manifestations (Niederer et al. 2019). Existing computational approaches have either used phenomenological organ-level descriptions without cellular calcium dynamics (Vossen et al. 2025) or detailed cellular electrophysiology without system-level hemodynamics (Campos et al. 2015), preventing determination of whether PESP patterns originate from intrinsic calcium handling or hemodynamic modulation.

This study addresses this gap by integrating calcium-mediated sarcomere mechanics into the existing CircAdapt framework (Van Osta et al. 2025), a closed-loop system model of the human heart and circulation. Building upon our previous phenomenological model (Vossen et al. 2025), we implement calcium dynamics including the calcium-mediated force–interval relationship (Yue et al. 1985) relevant for PESP generation while maintaining computational efficiency for future translational research applications. We pursue three specific aims: (1) validate that this approach reproduces experimental mechanical restitution curves, (2) determine the relative contributions of calcium dynamics versus loading conditions to PESP, and (3) examine why contractility metrics diverge in their assessment of post-extrasystolic responses. This mechanistic foundation is necessary for developing risk assessment tools in PVC-induced cardiomyopathy.

## Methods

### Model description

The simulations in this study were performed using the CircAdapt framework (www.circadapt.org), which models beat-to-beat cardiovascular mechanics and hemodynamics from cell to system level (Van Osta et al. 2025). CircAdapt includes modules representing cardiac myofibers, walls, valves, large blood vessels, systemic and pulmonary vasculature, and the pericardium (Arts et al. 2005; Lumens et al. 2009; Walmsley et al. 2015). This in silico environment enables precise variation of model parameters to accurately simulate both physiological and pathological conditions. For more detail, we refer the reader to the validation papers (Lumens et al. 2009; Walmsley et al. 2015; Van Osta et al. 2025).

CircAdapt is a lumped-parameter, closed-loop cardiovascular model that simulates beat-to-beat cardiovascular mechanics and hemodynamics from the sarcomere to the system level. The foundation of the model is the one-fiber model (Arts et al. 1991), which establishes relationships between cavity pressure and volume and myofiber stress and strain by assuming homogeneity of fiber stress and strain throughout the cardiac walls. The key geometric assumption is a thick-walled, rotationally symmetric geometry for the cardiac walls, with conservation of energy as the governing physical principle. Sarcomere mechanics are described using a mechanochemical contraction model (Arts et al. 2024), in which myofiber stress is the summation of active stress generated by actin-myosin cross-bridge formation and passive stress from elastic structures, primarily the extracellular matrix and titin. The atria are represented as spherical cavities surrounded by a spherical wall, while the ventricles are modeled using the TriSeg module (Lumens et al. 2009), in which the right and left ventricle share the interventricular septum and are in mechanical equilibrium at their hinge points. The circulatory system is represented by a closed-loop reduced-order model consisting of lumped systemic and pulmonary circulations, with large vessels described by a nonlinear pressure–area relation and microcirculatory flow governed by a nonlinear peripheral resistance. Mean arterial pressure and cardiac output are regulated by the Pressure Flow Control module, which adjusts peripheral resistance and total blood volume.

**Fig. 1:**
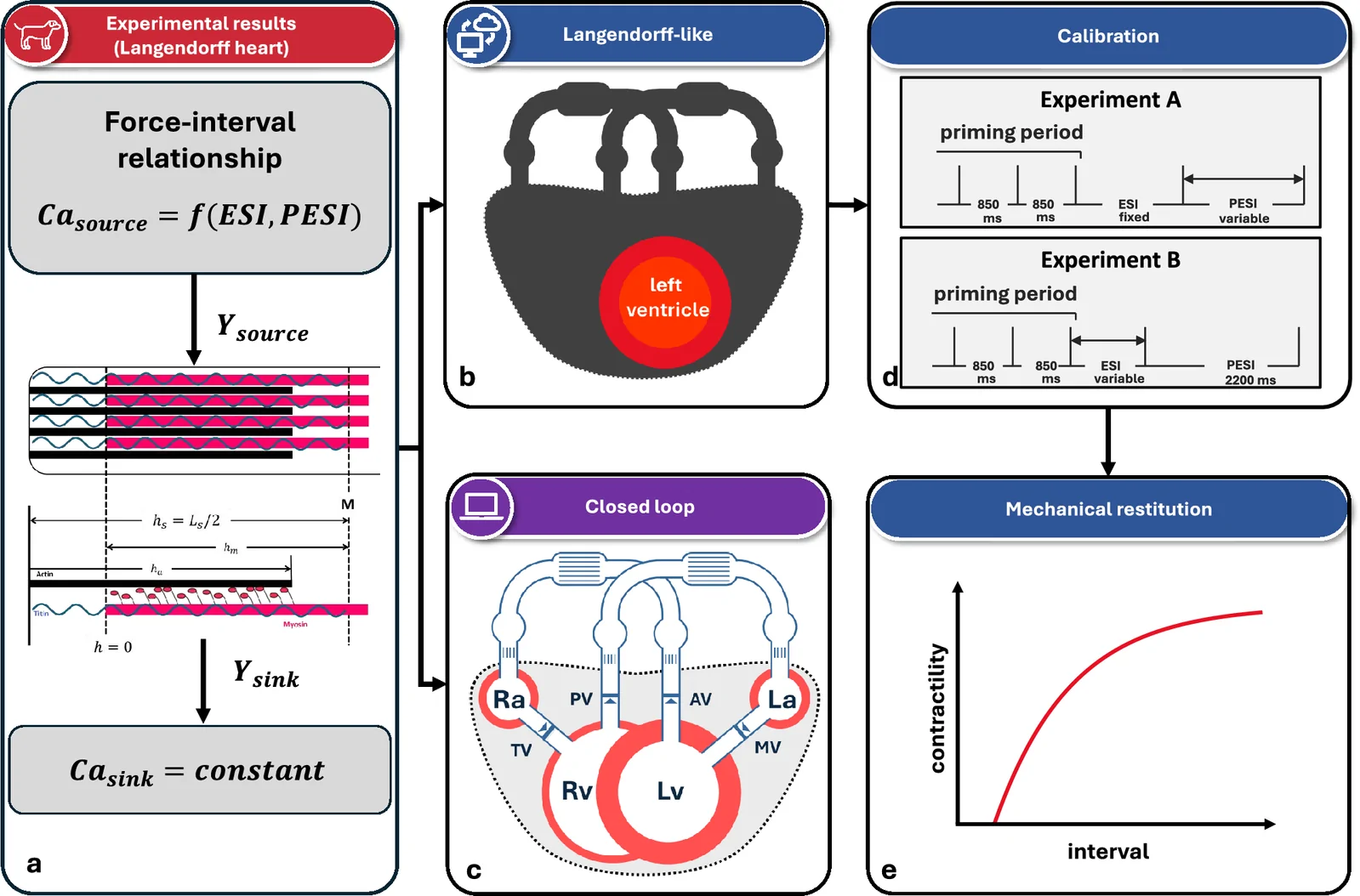
Global study overview. **a** A force–interval relationship (FIR), describing how contractile force depends on the timing of preceding beats, from Yue et al. (1985) is incorporated in a computational model of mechanochemical calcium-contraction dynamics (Arts et al. 2024). **b** Langendorff-like setup that resembles experimental conditions from the FIR and consists solely of the left ventricular (LV) cavity, and **c** the closed-loop CircAdapt model designed as a network of modules representing atrial and ventricular cardiac walls, blood vessels, and cardiac valves. **d** Virtual pacing experiments to create mechanical restitution curves (MRCs), where experiment A keeps the extrasystolic interval (ESI), the time between the last normal beat and the premature beat, constant while varying the post-extrasystolic interval (PESI), the time between the premature beat and the subsequent normal beat. Experiment B has a fixed PESI and varying ESI. Panel **e** shows the schematic of a mechanical restitution curve (MRC)

In this study, we used two CircAdapt-derived models. The first is a virtual Langendorff model of isolated left ventricular (LV) mechanics (see Fig. 1b), which replicates the experimental conditions described by Yue et al. (1985). This setup is characterized by an isolated LV cavity with constant preload and afterload, which enables detailed analysis of pressure–volume relationships and PESP conditions. The second model uses CircAdapt’s default closed-loop configuration (Walmsley et al. 2015) to represent the heart and circulatory system as a lumped parameter model, see Fig. 1c. This configuration enables study of how tissue-level changes affect organ-level function and overall circulatory system dynamics.

### Calcium-contraction coupling

The mechanochemical model by Arts et al. (2024) simulates myocardial contraction by coupling intracellular calcium dynamics to sarcomere mechanics through a biophysical cross-bridge interaction mechanism. It uses calcium-dependent activation and force generation that inherently account for physiological length dependences, such as the Frank–Starling mechanism, by linking sarcomere length, passive stiffness, and active tension generation. As a result, the model naturally reproduces key mechanical behaviors such as preload sensitivity and load-dependent relaxation due to its structure-based design. The sarcomere mechanics model is parameterized on data from multiple species, including rat, human, and murine measurements (Kentish et al. 1986). Within CircAdapt, the same model has been used to reproduce invasively measured left ventricular pressure–volume behavior in human cohorts with hypertrophic cardiomyopathy and HFpEF, correctly distinguishing increased chamber resting tone as a determinant of diastolic dysfunction against clinically recorded pressure–volume data (Tamargo et al. 2023).

All equations governing sarcomere active and passive stress and cross-bridge kinetics used in this study are adopted unchanged from Arts et al. (2024). The only modification is to ${\mathrm{Ca}}_{\mathrm{s}\mathrm{o}\mathrm{u}\mathrm{r}\mathrm{c}\mathrm{e}}$, which in Arts et al. (2024) is a fixed constant and is here replaced by a phenomenological function of the extrasystolic and post-extrasystolic intervals, governing the force–interval relationship, introduced below.

This electrophysiological system of calcium is connected to a source and sink via conductance. The sarcomere is connected continuously to a calcium sink with normalized concentration ${\mathrm{Ca}}_{\mathrm{s}\mathrm{i}\mathrm{n}\mathrm{k}}$ through a calcium conductance ${Y}_{\mathrm{s}\mathrm{i}\mathrm{n}\mathrm{k}}$, see Fig. 1a. During the time interval ${\tau}_{\mathrm{a}\mathrm{c}\mathrm{t}}$ of activation, the sarcomere is connected to a calcium source with normalized concentration ${\mathrm{Ca}}_{\mathrm{s}\mathrm{o}\mathrm{u}\mathrm{r}\mathrm{c}\mathrm{e}}$ with conductance ${Y}_{\mathrm{s}\mathrm{o}\mathrm{u}\mathrm{r}\mathrm{c}\mathrm{e}}$. The total amount of attached cross-bridges (Xb), as indicated by $\mathrm{X}\mathrm{b}$, is assumed to be proportional with the amount of calcium bound to Troponin. Therefore, for the time derivative of $\mathrm{X}\mathrm{b},$ we use:

1 $$\frac{\mathrm{d}Xb}{\mathrm{d}t}={\mathrm{Switch}}\left(t,{\uptau}_{\mathrm{a}\mathrm{c}\mathrm{t}}\right){Y}_{\mathrm{s}\mathrm{o}\mathrm{u}\mathrm{r}\mathrm{c}\mathrm{e}}\left({\mathrm{Ca}}_{\mathrm{s}\mathrm{o}\mathrm{u}\mathrm{r}\mathrm{c}\mathrm{e}}-{\mathrm{Ca}}_{\mathrm{e}\mathrm{q}}\right)+{Y}_{\mathrm{s}\mathrm{i}\mathrm{n}\mathrm{k}}\left({\mathrm{Ca}}_{\mathrm{s}\mathrm{i}\mathrm{n}\mathrm{k}}-{\mathrm{Ca}}_{\mathrm{e}\mathrm{q}}\right)$$

The function $Switch\left(t,{\uptau}_{\mathrm{a}\mathrm{c}\mathrm{t}}\right)$ equals 1 in time interval $0<t<{\uptau}_{\mathrm{a}\mathrm{c}\mathrm{t}}$ and equals zero during the rest of the time. It is known that the above exchange of calcium is a simplified model that prescribes influx and efflux of sarcomere calcium, rather than incorporating the known complexity of intracellular calcium handling.

While Eq. 1 does not contain an explicit velocity term, shortening rate dependence is incorporated implicitly through the tension feedback loop (Arts et al. 2024): Active tension feeds back onto the thick filament equilibrium, shifting Xb attachment toward the detached state during shortening. This reproduces the well-known force–velocity relationship without requiring an explicit velocity-dependent rate in the $\mathrm{d}\mathrm{X}\mathrm{b}/\mathrm{d}t$ formulation.

### Force–interval relationship

The coupling interval (CI) is defined here as the time elapsed from the onset of the last normal sinus beat to the onset of the premature beat (de Vries et al. 2018). Mechanical restitution (MR) refers to the phenomenon whereby the contractile strength of a beat depends on the duration of the preceding diastolic interval: A longer interval permits more complete sarcoplasmic reticulum calcium refilling, resulting in a stronger subsequent contraction, see Fig. 1e (Pásek and Nováková 2025). The force–interval relationship (FIR) describes how post-extrasystolic contractile strength varies with the extrasystolic interval (ESI, the interval between previous ventricular contraction and early ventricular contraction), and post-extrasystolic interval (PESI, the interval between premature ventricular contraction and next normal ventricular contraction), see Fig. 1d. Contractility is quantified here as the maximum rate of systolic left ventricular pressure rise, max(d*P*_Lv_/d*t*), where max() denotes the temporal peak during systole, and the subscript Lv identifies left ventricular pressure. This is expressed as a normalized ratio: max(d*P*_Lv_/d*t*)_PES_ / max(d*P*_Lv_/d*t*)_SS_ × 100%, where the subscripts PES and SS denote the post-extrasystolic beat and steady-state beat, respectively (Schmidt et al. 1995). Experiments performed on an isolated canine Langendorff heart setup have shown that an inverse relationship is present between ESI and PESP, while keeping the PESI constant (Yue et al. 1985). Furthermore, a positive relationship was present between PESI and PESP, while keeping the ESI constant.

We start with the formulation given by Yue et al. (1985), where normalized maximum rate of pressure rise during the post-extrasystolic beat is assumed to be related to the extrasystolic (ES) and post-extrasystolic (PES) duration. This relation is given by:

2 $$\frac{{\mathrm{max}\left(\frac{\mathrm{d}{P}_{Lv}}{dt}\right)}_{\mathrm{PES}}}{{\mathrm{max}\left(\frac{\mathrm{d}{P}_{Lv}}{\mathrm{d}t}\right)}_{\mathrm{SS}}}=\underbrace{\left\{A\cdot \mathrm{exp}\left[-\frac{\mathrm{ESI}-{t}_{0,\mathrm{es}}}{{\tau}_{\mathrm{mrc}}}\right]+B\right\}}_{\mathrm{ES-term}}\cdot \underbrace{\left\{1-\mathrm{exp}\left[-\frac{\mathrm{PESI}-{t}_{0,\mathrm{pes}}}{{\tau}_{\mathrm{mrc}}}\right]\right\}}_{\mathrm{PES-term}}$$

Here A and B are scaling parameters, ${\tau}_{\mathrm{m}\mathrm{r}\mathrm{c}}$ is the time constant governing MR, and ${t}_{0,es}$ and ${t}_{0,\mathrm{p}\mathrm{e}\mathrm{s}}$ are the intercepts with the horizontal axis for the MR curves. Since the model is calibrated in a Langendorff-like setup, where preload and afterload are kept constant, we assume that the change in contractility can only originate from an increase in calcium release:

3 $$\frac{{\mathrm{max}\left(\frac{\mathrm{d}{P}_{Lv}}{\mathrm{d}t}\right)}_{\mathrm{P}\mathrm{E}\mathrm{S}}}{{\mathrm{max}\left(\frac{\mathrm{d}{P}_{Lv}}{\mathrm{d}t}\right)}_{\mathrm{S}\mathrm{S}}}(\mathrm{E}\mathrm{S}\mathrm{I}, \mathrm{P}\mathrm{E}\mathrm{S}\mathrm{I})=\frac{{\mathrm{Ca}}_{\mathrm{s}\mathrm{o}\mathrm{u}\mathrm{r}\mathrm{c}\mathrm{e}}}{{\mathrm{Ca}}_{0}}$$

where ${\mathrm{Ca}}_{\mathrm{s}\mathrm{o}\mathrm{u}\mathrm{r}\mathrm{c}\mathrm{e}}(\mathrm{E}\mathrm{S}\mathrm{I},\mathrm{P}\mathrm{E}\mathrm{S}\mathrm{I})$ is the increase in calcium release, relative to the baseline release ${\mathrm{Ca}}_{0}$. Additionally, the ES term from Eq. 2 can be rewritten as follows:

4 $$\begin{array}{l}A\cdot \mathrm{exp}\left[-\frac{\mathrm{ESI}-{t}_{0,\mathrm{es}}}{{\tau}_{\mathrm{mrc}}}\right]+B=A\cdot \mathrm{exp}\left[-\frac{\mathrm{ESI}}{{\tau}_{\mathrm{mrc}}}+\frac{{t}_{0,\mathrm{es}}}{{\tau}_{\mathrm{mrc}}}\right]+B\\ \hphantom{A\cdot \mathrm{exp}\left[-\frac{\mathrm{ESI}-{t}_{0,\mathrm{es}}}{{\tau}_{\mathrm{mrc}}}\right]+B}=A\cdot \mathrm{exp}\left[-\frac{\mathrm{ESI}}{{\tau}_{\mathrm{mrc}}}\right]\mathrm{exp}\left[\frac{{t}_{0,\mathrm{es}}}{{\tau}_{\mathrm{mrc}}}\right]+B\end{array}$$

Since $\mathrm{exp}\left[\frac{{t}_{0,\mathrm{e}\mathrm{s}}}{{\tau}_{\mathrm{m}\mathrm{r}\mathrm{c}}}\right]$ is constant (e.g., not dependent on ESI and PESI), this can be included in constant A, resulting in:

5 $$\begin{array}{l}\frac{{\mathrm{max}\left(\frac{\mathrm{d}{P}_{Lv}}{\mathrm{d}t}\right)}_{\mathrm{PES}}}{{\mathrm{max}\left(\frac{\mathrm{d}{P}_{Lv}}{\mathrm{d}t}\right)}_{\mathrm{SS}}}=\left\{A\cdot \mathrm{exp}\left[-\frac{\mathrm{ESI}}{{\tau}_{\mathrm{mrc}}}\right]+B\right\}\\ \hphantom{\frac{{\mathrm{max}\left(\frac{\mathrm{d}{P}_{Lv}}{\mathrm{d}t}\right)}_{\mathrm{PES}}}{{\mathrm{max}\left(\frac{\mathrm{d}{P}_{Lv}}{\mathrm{d}t}\right)}_{\mathrm{SS}}}=}\cdot \left\{1-\mathrm{exp}\left[-\frac{\mathrm{PESI}-{t}_{0,\mathrm{pes}}}{{\tau}_{\mathrm{mrc}}}\right]\right\}\end{array}$$

Since for a steady beat should hold $\mathrm{E}\mathrm{S}\mathrm{I}=\mathrm{P}\mathrm{E}\mathrm{S}\mathrm{I}=\mathrm{S}\mathrm{S}\mathrm{I}$ (where SSI is the steady-state interval), it should give no change in contractility (e.g., $\frac{{\mathrm{max}\left(\frac{\mathrm{d}{P}_{Lv}}{\mathrm{d}t}\right)}_{\mathrm{P}\mathrm{E}\mathrm{S}}}{{\mathrm{max}\left(\frac{\mathrm{d}{P}_{Lv}}{\mathrm{d}t}\right)}_{\mathrm{S}\mathrm{S}}}=1$):

6 $$\left\{A\cdot \mathrm{exp}\left[-\frac{\mathrm{S}\mathrm{S}\mathrm{I}}{{\tau}_{\mathrm{m}\mathrm{r}\mathrm{c}}}\right]+B\right\}\left\{1-\mathrm{exp}\left[-\frac{\mathrm{S}\mathrm{S}\mathrm{I}-{t}_{0,\mathrm{p}\mathrm{e}\mathrm{s}}}{{\tau}_{\mathrm{m}\mathrm{r}\mathrm{c}}}\right]\right\}=1$$

Solving for B gives:

7 $$B=1/\left(1-\mathrm{exp}\left[-\frac{\mathrm{S}\mathrm{S}\mathrm{I}-{t}_{0,\mathrm{p}\mathrm{e}\mathrm{s} }}{{\tau}_{\mathrm{m}\mathrm{r}\mathrm{c}}}\right] \right)-A\cdot \mathrm{exp}\left[-\frac{\mathrm{S}\mathrm{S}\mathrm{I}}{{\tau}_{\mathrm{m}\mathrm{r}\mathrm{c}}}\right]$$

This gives the final expression:

$$\begin{array}{l}{\mathrm{Ca}}_{\mathrm{source}}\left(\mathrm{ESI},\mathrm{PESI}\right)={\mathrm{Ca}}_{0}\left(A\cdot \mathrm{exp}\left[\frac{-\mathrm{ESI}}{{\tau}_{\mathrm{mrc}}}\right]+B\right)\\ \hphantom{{\mathrm{Ca}}_{\mathrm{source}}\left(\mathrm{ESI},\mathrm{PESI}\right)=}\cdot \left(1-\mathrm{exp}\left[\frac{-\left(\mathrm{PESI}-{t}_{0,\mathrm{pes}}\right)}{{\tau}_{\mathrm{mrc}}}\right]\right)\end{array}$$

8 $$\mathrm{w}\mathrm{i}\mathrm{t}\text{h } B=1/\left(1-\mathrm{exp}\left[-\frac{\mathrm{S}\mathrm{S}\mathrm{I}-{t}_{0,\mathrm{p}\mathrm{e}\mathrm{s} }}{{\tau}_{\mathrm{m}\mathrm{r}\mathrm{c}}}\right] \right)-A\cdot \mathrm{exp}\left[-\frac{\mathrm{S}\mathrm{S}\mathrm{I}}{{\tau}_{\mathrm{m}\mathrm{r}\mathrm{c}}}\right]$$

Constant B is chosen such that when no PVC occurs, it holds that $\mathrm{E}\mathrm{S}\mathrm{I}=\mathrm{P}\mathrm{E}\mathrm{S}\mathrm{I}=\mathrm{S}\mathrm{S}\mathrm{I}$, which results in ${\mathrm{Ca}}_{\mathrm{s}\mathrm{o}\mathrm{u}\mathrm{r}\mathrm{c}\mathrm{e}}={\mathrm{Ca}}_{0},$ and no change in calcium release is observed.

### Simulations

#### Validation using mechanical restitution

The novel introduced FIR was calibrated using experimental data from Yue et al. (1985). This study relates the isolated, isovolumic beating canine left ventricle to a description of post-extrasystolic contractile strength (max(dP_Lv_/dt)) as a function of extrasystolic and post-extrasystolic stimulus intervals. Both intervals are varied over a physiological range in this dataset, and this two-dimensional sampling of the (ESI, PESI) space is required to identify the two-term formulation. While the original experiments were conducted in dogs with a baseline cycle time of 460 ms, human baseline cycle times are approximately 850 ms. The pacing protocol is, therefore, scaled by a factor of ~ 1.8 to match human heart rate and pacing ranges.

The same simulation protocols were used as described in Vossen et al. (2025). Briefly, MRCs were generated using the Virtual Langendorff model, applying pacing protocols from experiments A and B (see Fig. 1d), where experiment A uses a fixed ESI (300, 350, 460, and 1200 ms) with variable PESI up to 2200 ms, and experiment B uses a fixed PESI (2200 ms) with a variable ESI up to 2200 ms. Change in max(dP_Lv_/dt) for the post-extrasystolic beat and extrasystolic beat is validated against experimental results with and without FIR.

The parameters A, ${\tau}_{\mathrm{m}\mathrm{r}\mathrm{c}},$ and ${t}_{0,\mathrm{p}\mathrm{e}\mathrm{s}}$ were estimated using a Monte Carlo-based approach to justify both the choice of optimization strategy and the uniqueness of the solution. An initial broad Monte Carlo sampling (n = 5000) was performed over wide parameter bounds to explore the global structure of the objective function. This sampling revealed a single well-defined minimum, indicating the presence of a unique global optimum. A second, more focused sampling (n = 1000) within a reduced parameter region confirmed this result and provided a refined estimate of the optimal parameter set. Based on this outcome, gradient-based optimization using the trust-region Newton conjugate gradient method (trust-ncg) was applied, initialized within the region identified by the Monte Carlo sampling. This ensured convergence from a starting point close to the global solution while relying on the prior sampling to establish uniqueness of the optimum.

The trust-ncg algorithm minimizes the sum of squared differences between simulated and experimental mechanical restitution curves. To ensure the uniqueness of the solution, a multi-start approach is applied during optimization, where the initial guess is randomly chosen within the bounds provided by the Monte Carlo sampling. More details about the optimization process are given in Supplementary file A.

Starting with the calibrated FIR, a quadrigeminy pattern with every fourth beat a PVC is simulated in a closed-loop CircAdapt model. The resulting hemodynamics, including LV pressure tracings and pressure–volume loops, are compared with clinical data from Brener et al. (2021). During this comparison, the MR calibration is kept constant, ensuring that a single parameter set described the entire behavior observed in the fully coupled model and links the calcium-level FIR to system-level dynamics. The coupling interval and compensatory pause were taken from the clinical trace, while the mechanical restitution calibration was held fixed. No parameters of the contraction model were adjusted, only one to better match the patient-specific cardiac geometry, so the comparison reflects the qualitative pattern produced by a generic healthy subject sharing only the beat timing of the clinical case.

Furthermore, the calibrated FIR was used in the same closed-loop CircAdapt model to evaluate the effect of different levels of prematurity. The default implementation of the CircAdapt model represents a healthy virtual subject with normal values for cardiac output (CO, 5.1 L/min), mean arterial pressure (MAP, 91.5 mmHg), heart rate (HR, 70 bpm), and the maximum rate of pressure rise in the left ventricle during systole (max(dP_Lv_/dt), 1631 mmHg s^−1^). Increased prematurity of PVCs was simulated by varying the coupling interval (CI) from 850 ms (no PVC) to 500 ms.

#### The role of loading conditions in PESP generation

To investigate how PESP depends on loading conditions and tissue properties, we systematically varied preload, afterload, and the linear active stress component in the closed-loop model. Preload and afterload were modulated via the pressure–flow control (PFC) module by adjusting target flow (CO = 5.1 L/min) and target pressure (MAP = 91.5 mmHg) by 50%, through tuning effective circulating blood volume and systemic peripheral resistance. Active tissue properties were varied by reducing the active stress contribution by 20%, simulating reduced contractility as seen in systolic dysfunction. For each condition, we simulated PVCs with coupling intervals ranging from 550 to 850 ms. PESP was quantified by comparing the post-extrasystolic beat to baseline sinus rhythm using three metrics: change in left ventricular ejection fraction (ΔLVEF), change in maximum rate of left ventricular pressure rise (∆max(dP_Lv_/dt)), and change in systolic blood pressure (ΔSBP).

#### Numerical implementations

All simulations were performed using the CircAdapt Framework version 2503. The core components of CircAdapt are implemented in C++ and accessed through a Python interface (Van Osta et al. 2025). All additional code was written and executed in Python 3.9.21. We used the default configuration as shown in previous work from Arts et al. (2024). Numerical computations and model integrations utilized SciPy 1.13.1 and NumPy 2.0.2, providing efficient handling of array operations and matrix calculations. The code used for producing these results is available at < https://github.com/CircAdapt/Vossen2026ForceIntervalRelationship > .

## Results

### Calibration of mechanical restitution

**Fig. 2:**
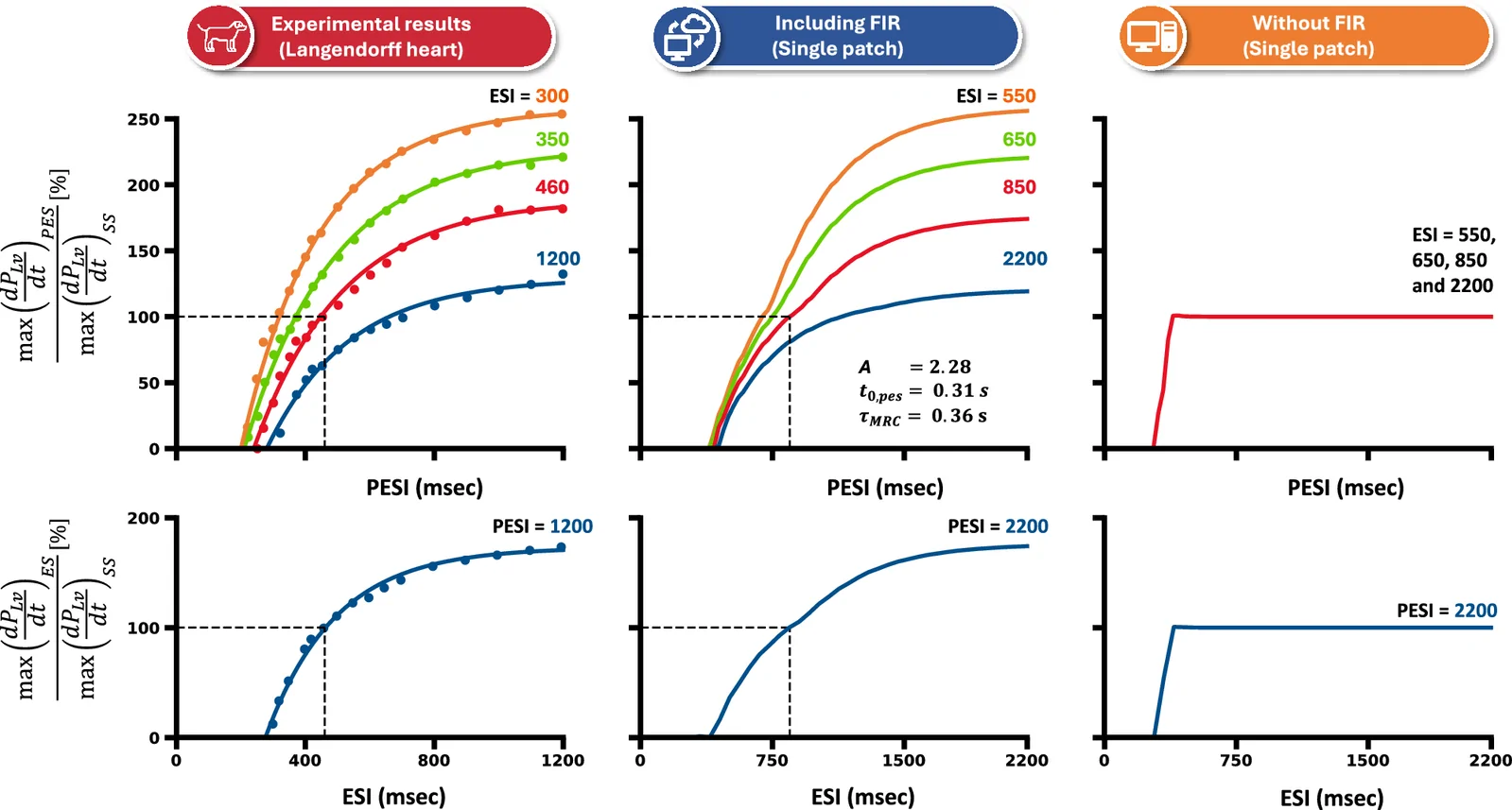
Experimental mechanical restitution curves (MRCs), describing the dependence of contractile strength on preceding beat intervals, (left column) versus simulated MRCs (middle column) for both experiment A (top row), in which the extrasystolic interval (ESI) is fixed and the post-extrasystolic interval (PESI) is varied, and experiment B (bottom row), in which the PESI is fixed and the ESI is varied. The right column shows results for the same experimental protocols without the force–interval relationship (FIR). The y-axis shows the peak rate of rise of left ventricular pressure, max(dP_Lv_/dt), normalized to the steady-state value. Experimental results are redrawn based on data from Yue et al. (1985)

Figure 2 shows the MR curves for both experiments A (top row) and B (bottom row). The experimental results are redrawn based on data from Yue et al. (1985) for reference. The simulated MRCs show good resemblance to the experimental MRCs. The plateau values of the curves agree, and the leftward shift of the curves for a smaller ESI is also present. The right panels show the change in max(dP_Lv_/dt) for the post-extrasystolic beat (above) and the extrasystolic beat (below) without the FIR. It shows that the contractility for both plots increases linearly to 100%, after which it reached its maximum and does not increase any further. Furthermore, except for the post-extrasystolic beat, the ESI does not influence the change in max(dP_Lv_/dt) as a function of PESI. These results validate the calcium-based force–interval implementation.

First iteration of Monte Carlo sampling showed a global minimum for samples with a RMSE smaller than 11.4%. This resulted in a second iteration of Monte Carlo sampling with reduced bounds, which resulted in one parameter set with a RMSE of 11.4%. Further gradient-based optimization using a multi-start (normal distribution, SD = 0.3) trust-ncg algorithm resulted in parameters A=2.35±0.10, ${\tau}_{\mathrm{m}\mathrm{r}\mathrm{c}}=0.36\pm 0.01$ s, and ${t}_{0,\mathrm{p}\mathrm{e}\mathrm{s}}=0.32\pm 0.01$ s with a RMSE of $9.59 \pm 0.08\%$. The final optimal parameter set was selected as the run achieving the lowest RMSE, yielding A = 2.28, ${\tau}_{\mathrm{m}\mathrm{r}\mathrm{c}}$ = 0.36 s, ${t}_{0,\mathrm{p}\mathrm{e}\mathrm{s}}$= 0.31 s, and B = 1.08 (derived from the steady-state constraint), with a final RMSE of 9.54%.

**Fig. 3:**
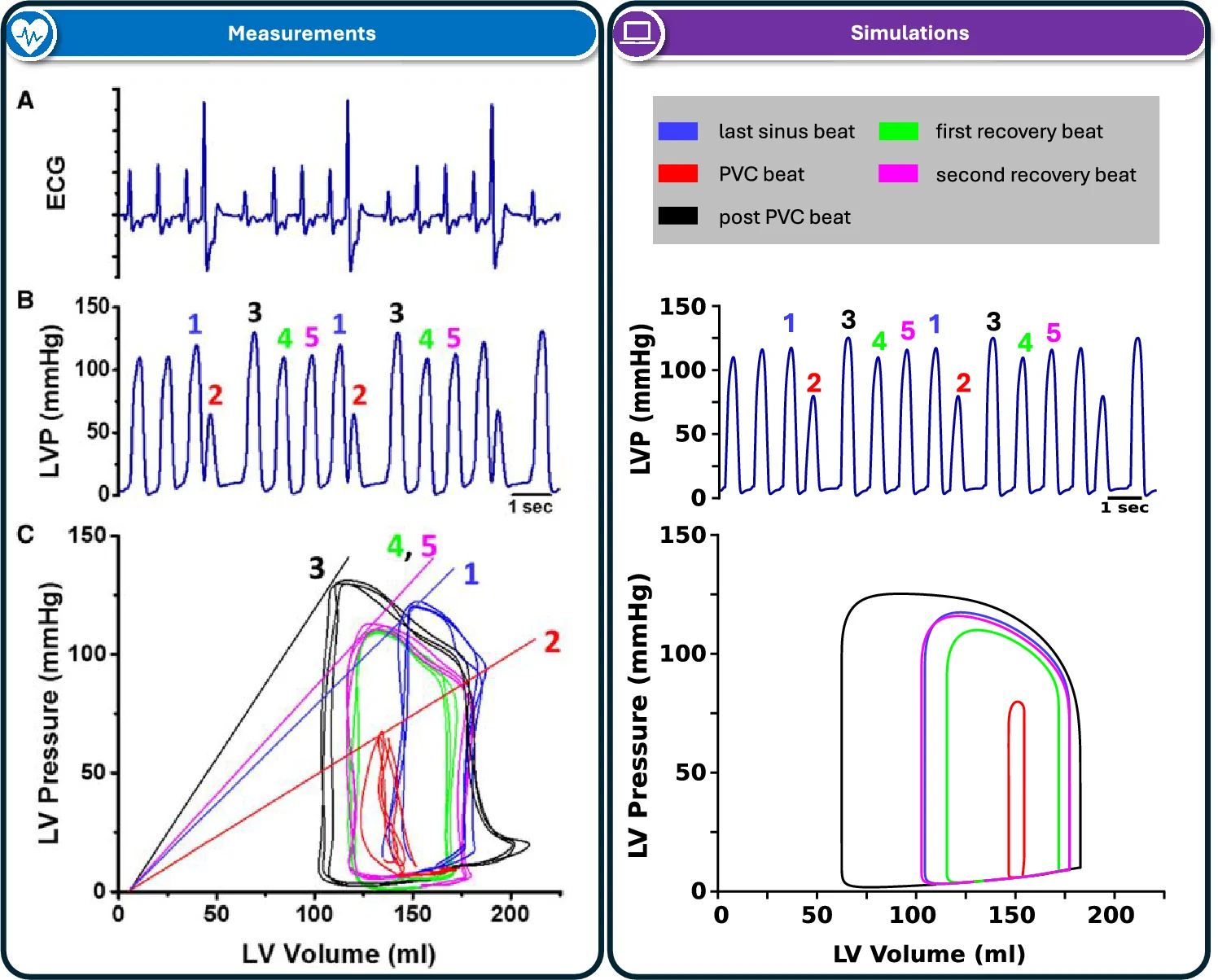
Comparison between clinical measurements (left) and simulations (right) of a quadrigeminy, a rhythm pattern in which every fourth beat is a premature ventricular complex (PVC). Row A shows the electrocardiogram (ECG) signal, row B shows left ventricular (LV) pressure tracings, and row C shows pressure–volume loops. Pressure–volume loops correspond to beats 1, 2, 3, 4, and 5 as labeled in row B, where beat 1 is the last normal sinus beat, beat 2 is the PVC, beat 3 is the post-extrasystolic beat showing post-extrasystolic potentiation (PESP), and beats 4 and 5 are recovery beats. Figure left reproduced with permission from Brener et al. (2021)

Figure 3 illustrates system-level PVC effects through pressure–volume loops and LV pressure tracings, obtained by simulating the PVC sequence with the coupling interval and compensatory pause obtained from Brener et al. (2021). A conductance catheter seated in the LV apex was used to assess changes in ventricular contractility via pressure–volume analysis, where a quadrigeminy pattern is measured (panel A). After the last sinus beat (1), a PVC (2) is measured, after which the post-PVC beat (3) shows a PESP. Beats 4 and 5 are recovery beats. The pressure–volume loops show the beat-to-beat contractility, as indicated by the changing slopes of the end-systolic elastance, of the respective end-systolic pressure–volume relationships.

Comparing the measurements with simulations, we can appreciate the agreement in LV pressure between the PVC beat (2) and the last normal sinus beat (1). Furthermore, the subsequent post-PVC beat (3) shows an increase in magnitude in both simulation and measurement results, demonstrating PESP. After this beat, two recovery beats follow.

Pressure–volume loops also demonstrate good qualitative agreement. The PVC beat shows a decreased SV with respect to the last normal sinus beat. Furthermore, the post-PVC shows increased filling, resulting in a larger end-diastolic volume (EDV), and a lower end-systolic volume (ESV) due to larger contractility.

**Fig. 4:**
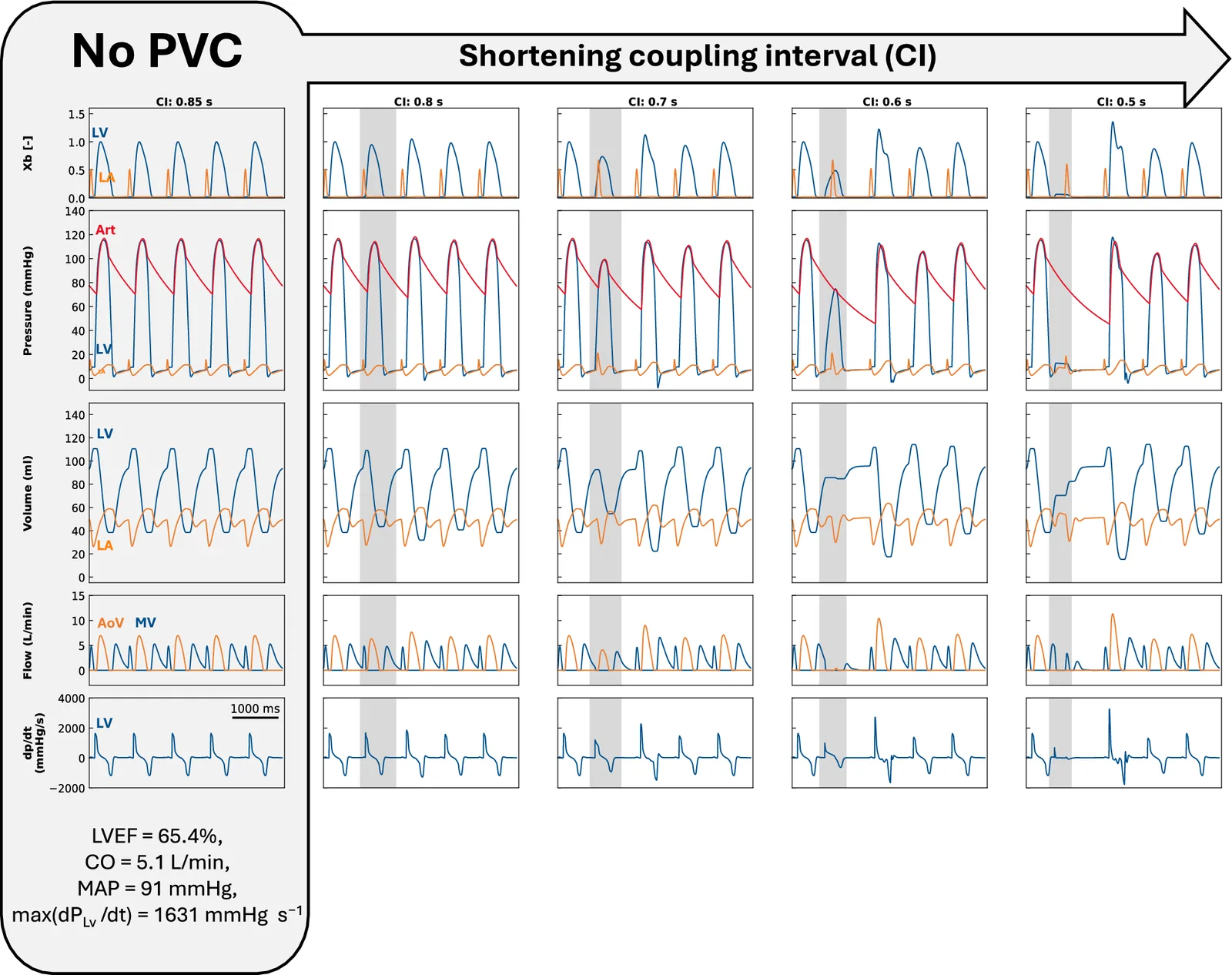
Effect of shortening the coupling interval (CI), defined as the time from the onset of the last normal sinus beat to the onset of the premature ventricular complex (PVC), on normalized cross-bridge formation (Xb) with respect to baseline, left ventricular (LV) pressure, LV volume, blood flow, and the peak rate of rise of LV pressure max(dP_Lv_/dt). The left panel shows the baseline simulation with no PVC. Increasing the level of prematurity from left to right results in decreased cross-bridge formation during the PVC beat and increased cross-bridge formation during the post-extrasystolic beat, producing a more prominent post-extrasystolic potentiation (PESP). LV: left ventricle; La: left atrium; Art: arterial system; AoV: aortic valve; MV: mitral valve; left ventricular ejection fraction (LVEF); cardiac output (CO); and mean arterial pressure (MAP). Gray areas indicate the PVC beat

Figure 4 shows the effects of shortening the CI on cardiac mechanics and hemodynamics during PVCs in the closed-loop model. The columns represent simulations with progressively shorter CI, from left to right, with the first column showing normal sinus rhythm (no PVC). Shorter coupling intervals produce coordinated hemodynamic changes: reduced Xb during the PVC, decreased LV systolic pressure (potentially preventing aortic valve opening), and subsequent increases in EDV during the compensatory pause. The enhanced post-PVC beat reflects combined effects of increased preload, reduced afterload, and calcium-mediated contractility enhancement. After the post-extrasystolic beat, the pressure gradually returns to baseline.

EDV increases after short-coupled PVCs due to the compensatory pause, leading to enhanced stroke volume in the following beat. The LV volume during the PVC beat is reduced when the contraction occurs prematurely. Aortic flow decreases during early coupled PVC beats as they may become non-ejecting. Mitral flow shows increased filling during the compensatory pause, supporting the subsequent enhanced LV ejection.

### The role of loading conditions in PESP generation

**Fig. 5:**
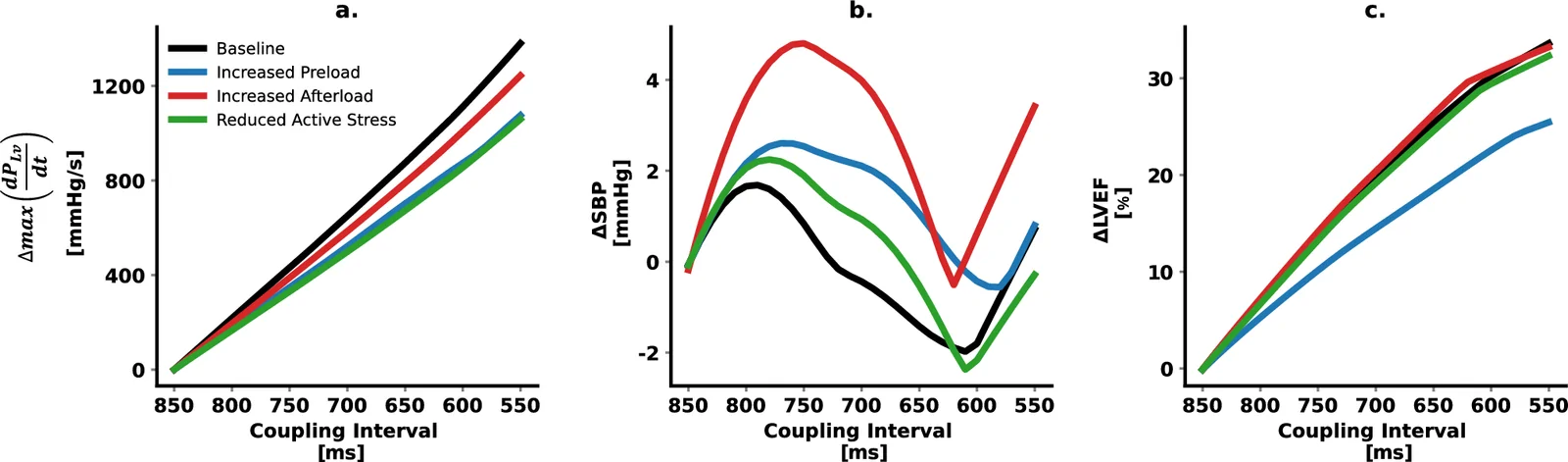
Relative change in contractility of the post-extrasystolic beat compared with the last normal sinus beat, expressed as the change in peak rate of the left ventricular pressure rise (∆max(dP_Lv_/dt)), change in systolic blood pressure (∆SBP), and change in the left ventricular ejection fraction (∆LVEF), shown across a range of coupling intervals (CI) for a single premature ventricular complex (PVC). Results are shown for baseline conditions, increased afterload (50%), increased preload (50%), and decreased active fiber tension (20%)

Having demonstrated that our calcium-based model reproduces experimental MR curves and reveals the mechanistic basis of PESP through Xb dynamics, we next examined how these mechanisms manifest under clinically relevant conditions. Figure 5 shows the effect of decreasing coupling interval on ∆LVEF, ∆SBP, and ∆max(dP_Lv_/dt), where Δ indicates the difference between post-extrasystolic beat and last normal sinus beat. Results are shown for baseline conditions (black line), increased afterload (red), increased preload (blue), and decreased active fiber tension (green).

Across all conditions, shorter coupling intervals led to progressively altered post-extrasystolic hemodynamics (Fig. 5a). ∆max(dP_Lv_/dt) consistently increased with shorter CI, with loading conditions providing only minor modulation. In contrast, ∆SBP exhibited complex non-monotonic behavior peaking at CI = 790ms for baseline conditions, with peaks shifting to shorter CI values under altered loading. ∆LVEF showed similar trends to ∆max(dP_Lv_/dt), though preload effect became more pronounced at shorter CI.

## Discussion

In this study, we integrated calcium-mediated sarcomere mechanics into the CircAdapt framework to investigate the mechanistic basis of post-extrasystolic potentiation. The model reproduced experimental force–interval relationships with high accuracy, demonstrating that our calcium implementation captures mechanical restitution. Our simulations show that calcium-mediated cross-bridge formation reproduces PESP within this model, with loading conditions modulating the magnitude and pattern of the response. We observed differential responses between contractility metrics: max(dP_Lv_/dt) consistently reflected calcium changes across loading conditions, while systolic blood pressure showed variable, load-dependent responses. These findings suggest that heterogeneous clinical outcomes in patients with similar PVC burdens may reflect individual differences in the balance between intrinsic calcium handling and extrinsic hemodynamic factors.

### Introducing the force–interval relationship in CircAdapt

Our simulations using the Langendorff-like model (Fig. 2) reveal key relationships between the timing of premature and subsequent beats and their effect on contractility. Specifically, a longer PESI allows for a larger increase in max(dP_Lv_/dt). Conversely, shortening the ESI leads to a more premature contraction with decreased contractility, but increases the potentiation of the following beat. These findings are consistent with the experimental observations reported by Yue et al. (1985), reinforcing the hypothesis that accurate representation of calcium handling is critical for reproducing PVC-induced hemodynamics. Previously, we demonstrated that directly modulating contractility based on the empirical relationship described by Yue et al. (1985) could reproduce key hemodynamic features of PVCs (Vossen et al. 2025). By integrating a calcium source model that inherently reflects this relationship, the current study provides further evidence that calcium dynamics play a central role in governing the mechanical response following premature beats.

The underlying calcium model (**Eqs. **(1) and (8)) captures the essential mechanisms of calcium-induced calcium release (CICR) using a lumped representation. Calcium influx through L-type calcium channels (LTCC) triggers sarcoplasmic reticulum (SR) calcium release via ryanodine receptors (RyR), initiating contraction, whereas relaxation follows as calcium is re-sequestered into the SR by SERCA and partially extruded via the sodium–calcium exchanger (NCX) (Bers 2002). In the context of PVCs, the compensatory pause provides sufficient time for SR calcium refilling, thereby intensifying the next contraction (Sprenkeler and Vos 2016). Although the current model successfully replicates this behavior, it remains a simplification. Future studies should investigate how well this simplification captures the essential features of PVC physiology and whether it is sufficient for studying the resulting hemodynamic effects, or if more detailed models are required. Future extensions may include spatial calcium gradients or detailed ion-channel kinetics to further refine the mechanistic understanding of PVC-induced hemodynamic changes and to explicitly simulate the effects mentioned above. Studies have shown (Kadambi et al. 1996; Hoit et al. 1999; Iribe et al. 2006) that these underlying mechanisms play a crucial role in further investigation of calcium kinetics. Because calcium dynamics are represented through abstract source-sink terms rather than through explicit subcellular mechanisms, the model cannot attribute PESP to specific processes such as SR calcium load, SERCA kinetics, or LTCC-mediated calcium entry. Therefore, the model demonstrates that a calcium-based mechanism is of sufficient complexity to reproduce the observed PESP pattern and to address the specific aims of this study, but it does not demonstrate that calcium is the exclusive driver of PESP.

### Beat-to-beat variability in contractility

Simulated quadrigeminy patterns (Fig. 3) captured the complex beat-to-beat interplay between calcium-mediated contractility and loading conditions observed in clinical data. The model’s ability to reproduce these dynamics using a single calibrated parameter set linking cellular FIR to system-level hemodynamics is consistent with the proposed mechanistic framework.

The hemodynamic response of a single PVC is directly influenced by the coupling intervals, as shown in Fig. 4. As the coupling interval (CI) shortens the introduced FIR results in less calcium released, and consequently, fewer cross-bridges form, which reduces pressure during the extrasystolic beat. This lowers stroke volume and can lead to a non-ejecting PVC if aortic pressure is not overcome. In contrast, the post-extrasystolic beat benefits from a longer pause, which results in a larger calcium release allowing more cross-bridges to form, increasing contractility, preload, and ejection due to reduced afterload. This increase in contractility with respect to the last normal beat, the PESP, is characteristic for PVCs in seemingly healthy tissue (Billet et al. 2019; Brener et al. 2021) and is a potential predictor for patients who might develop PVC-induced cardiomyopathy (Dukes et al. 2015; Billet et al. 2019; Kowlgi et al. 2020).

The simulated and measured pressure–volume loops agree in their qualitative beat-to-beat structure but not in absolute volumes, most visibly in the pre-extrasystolic cycle. Several factors contribute to this. The simulation represents a generic healthy subject rather than a patient-specific reconstruction, so its operating volumes reflect the default model configuration rather than those of the individual patient, and the clinically measured volumes depend on the conductance-catheter calibration. In addition, the implemented force–interval relationship depends only on the two preceding intervals, whereas physiological restitution reflects a longer history of preceding beats. This two-beat memory may limit how well the pre-extrasystolic cycle of a repeating quadrigeminy can be reproduced.

### Coupling interval determines hemodynamic consequences

As the CI shortens, extrasystolic beats transition from ejecting to non-ejecting PVCs. In our simulations, this transition occurs at a CI of 630 ms, as shown in Fig. 5. There is not enough pressure generated to overcome afterload, and the aortic valve stays closed. While a further shortening of CI does not affect stroke volume in the non-ejecting PVCs, it increases the compensatory pause, which results in a more contractile beat. Furthermore, early rise in SBP around 800 ms can be attributed to the absence of A wave due to early contraction of the ventricle. After this, there is no active contribution to the filling of the ventricle, which, therefore, results in smaller filling pressures.

Beyond the single PVC and quadrigeminy scenarios, the model provides a framework for exploring how loading conditions reshape the post-extrasystolic response. Systemic variation of preload, afterload, and active tension (Fig. 5) showed that the three contractility metrics respond differently. $\Delta \mathrm{max}\left(\frac{\mathrm{d}{P}_{Lv}}{\mathrm{d}t}\right)$ increased as the coupling interval shortened and was only weakly modulated by loading, whereas Δ SBP was non-monotonic and strongly load-dependent, and Δ LVEF followed $\Delta \mathrm{max}\left(\frac{\mathrm{d}{P}_{Lv}}{\mathrm{d}t}\right)$ while becoming more preload sensitive at short coupling intervals. Because $\frac{\mathrm{d}P}{\mathrm{d}t}$ based metrics reflect predominantly the intrinsic, calcium-mediated component of potentiation while pressure-based metrics also include the loading state, their relationship in an individual patient may in principle carry information about the balance between intrinsic myocardial and extrinsic loading contributions to PESP. The persistence of PESP patterns across varied loading conditions is consistent with a calcium-mediated mechanism, with hemodynamics providing quantitative modulation. This distinction has clinical implications for interpreting PESP magnitude in patients with different loading states.

### Limitations and future outlook

Our lumped calcium model captures essential restitution behavior while maintaining computational efficiency for large-scale simulations. Although this approach cannot resolve detailed calcium cycling dynamics (e.g., beat-to-beat variability in transients and altered SERCA kinetics), such detail is unnecessary for studying PESP dynamics in both healthy and failing myocardium. More detailed electrophysiological models would be required to investigate specific subcellular mechanisms.

The components of the integrated model are parameterized on data obtained in different species. The restitution calibration on the isolated canine left ventricle (Yue et al. 1985), the sarcomere mechanics on rat, human, and murine measurements (Kentish et al. 1986; Arts et al. 2024), and the hemodynamic configuration on the healthy adult human. The consequence for interpretation is that the model reproduces the qualitative structure of the force–interval relationship and its propagation across scales, whereas absolute magnitudes carry correspondingly greater uncertainty and should not be read as species- or patient-specific quantitative predictions. Human force–interval measurements (Gwathmey et al. 1990) are qualitatively consistent with the present results, a shorter extrasystolic interval being associated with a more contractile post-extrasystolic beat. A fully human-parameterized calcium model, calibrated against controlled human myocardial restitution data, would reduce this uncertainty and improve the quantitative precision of patient-specific predictions; such calibration is currently limited by the scarcity of suitable human experimental measurements and represents an important direction for future work.

Although length-dependent calcium sensitivity is implicitly represented through the titin-mediated tension feedback pathway in the sarcomere mechanics model, the ${\mathrm{Ca}}_{\mathrm{s}\mathrm{o}\mathrm{u}\mathrm{r}\mathrm{c}\mathrm{e}}$ term is parameterized solely as a function of ESI and PESI and is not directly modulated by sarcomere length. Stretch-activated ion channels and other mechano-electric or mechano-calcium feedback pathways are similarly absent. The ${\mathrm{Ca}}_{\mathrm{s}\mathrm{o}\mathrm{u}\mathrm{r}\mathrm{c}\mathrm{e}}$ calibration was performed under isovolumic conditions using data from Yue et al. (1985), where sarcomere length does not vary between beats, so this omission does not affect the calibration itself. The available experimental data currently do not support a more complete, length-dependent parameterization. Sarcomere length does vary beat-to-beat in the closed-loop model, however, particularly through changes in end-diastolic volume associated with the compensatory pause, so the absence of these mechanically driven feedback pathways there means the magnitude of the simulated potentiation may be under- or overestimated relative to the intact circulation. More fundamentally, because these pathways are omitted, the model cannot discriminate between a purely calcium release-driven explanation of beat-to-beat force adaptation and one in which mechanically driven feedback also contributes.

Early ventricular contraction is modeled by simultaneous activation of both ventricles. In reality, premature ventricular contractions arise from an ectopic focus and propagate slowly through the myocardium, bypassing the fast conduction system. This produces heterogeneous activation, reduces mechanical efficiency, and alters the pressure–volume loop. The primary outcome in this study, post-extrasystolic potentiation is mainly governed by calcium handling in the post-extrasystolic beat. The compensatory pause depends on the extrasystolic and post-extrasystolic intervals, rather than the site of origin, so the modeled restitution mechanism remains qualitatively valid. However, the origin of the PVC does affect hemodynamics, with outflow tract PVCs generally better tolerated than apical PVCs, as shown by Baman et al. (2010). Future work could incorporate spatial activation patterns using an Eikonal model coupled to CircAdapt to assess how ectopic origin influences global hemodynamics and PESP magnitude.

PVCs engage systemic regulatory mechanisms beyond local cardiac mechanics. The baroreflex response to PVC-induced pressure drops manifests as heart rate turbulence (HRT), a short-term rhythm fluctuation mediated by autonomic reflexes (Schmidt et al. 1999; Bauer et al. 2008). Patients with heart failure show diminished HRT and blunted compensatory responses (Schmidt et al. 1999), suggesting that high PVC burdens may overwhelm baroreflex capacity, leading to maladaptive cardiovascular feedback (Baman et al. 2010; Dukes et al. 2015). Incorporating reflex pathways into future modeling efforts would provide more complete understanding of PVC pathophysiology in progressive disease states.

The comparison with the clinical recording of Brener et al. (2021) in Fig. 3 is presented as a single illustrative case, intended to show that the model reproduces the qualitative beat-to-beat pattern of a PVC sequence rather than providing quantitative validation. A prospective patient study should demonstrate the ability of this model to replicate PVC sequences and identify patients’ underlying tissue abnormalities.

Because ${\mathrm{Ca}}_{\mathrm{s}\mathrm{o}\mathrm{u}\mathrm{r}\mathrm{c}\mathrm{e}}$ is calibrated to data that already exhibit the PESP pattern, the model cannot independently confirm calcium as the primary mechanism. Rather, it demonstrates that a calcium-based formulation is consistent with the observations. Moreover, the conductance and concentration parameters of the calcium exchange model (${Y}_{\mathrm{s}\mathrm{o}\mathrm{u}\mathrm{r}\mathrm{c}\mathrm{e}}$, ${Y}_{\mathrm{s}\mathrm{i}\mathrm{n}\mathrm{k}}$, ${\mathrm{Ca}}_{\mathrm{s}\mathrm{i}\mathrm{n}\mathrm{k}}$, and ${\tau}_{\mathrm{a}\mathrm{c}\mathrm{t}}$) were set heuristically to reproduce human physiological hemodynamics rather than being independently constrained by experimental calcium measurements, which further limits the mechanistic specificity of the conclusions and should be considered when interpreting the quantitative aspects of the results. Loading conditions and calcium dynamics are intrinsically coupled in the intact circulation, and the relative contribution attributed to each depends in part on the assumptions embedded in the ${\mathrm{Ca}}_{\mathrm{s}\mathrm{o}\mathrm{u}\mathrm{r}\mathrm{c}\mathrm{e}}$ formulation.

## Conclusion

We developed a computational platform coupling calcium dynamics with whole-heart hemodynamics to examine post-extrasystolic potentiation mechanisms in premature ventricular complexes, demonstrating that a calcium-mediated mechanism is sufficient to explain the observed PESP patterns. Our findings indicate that a calcium-mediated force–interval mechanism reproduces the observed PESP patterns within this framework, with preload and afterload modulating the pattern of beat-to-beat pressure response. Future research should aim to use a more detailed calcium model to better differentiate between different contributors of PESP. The divergent behavior of max(dP_Lv_/dt) versus systolic blood pressure suggests that clinical assessment requires multiple contractility metrics. This framework provides a foundation for distinguishing intrinsic myocardial dysfunction from extrinsic loading effects, thereby facilitating patient-specific risk stratification for PVC-induced cardiomyopathy.

## Supplementary Information

Below is the link to the electronic supplementary material.

Supplementary file1 (DOCX 1348 kb)

## Data Availability

The code used for producing these results is available at < https://github.com/CircAdapt/Vossen2026ForceIntervalRelationship > .
