# Supplementary material for: Calcium-mediated force–interval relationship drives post-extrasystolic potentiation in premature ventricular complexes: a computational study
Source: Biomech Model Mechanobiol. 2026 Aug 30;25(5):96. doi: 10.1007/s10237-026-02119-w (PMC13526725; doi:10.1007/s10237-026-02119-w)
Supplement: Supplementary file 1 [file 10237_2026_2119_MOESM1_ESM.docx]

**SUPPLEMENTARY FILE A- MONTE-CARLO SAMPLING AND OPTIMIZATION**

To verify reproducibility of the parameter estimation described in the methods, the full optimization procedure is further elaborated in this appendix. The optimization process followed a successive three-step strategy. First, a broad Monte Carlo sampling was performed within wide parameter bounds: $A$ ∈ [0.10, 5.00], $\tau_{mrc}$ ∈ [0.01, 2.00] s, and $t_{0,pes}$∈ [0.00, 0.84] s. A total of 5000 samples was evaluated in this first stage, with all 5000 samples yielding a finite RMSE. The best RMSE obtained in this stage was 11.4%. The resulting distribution of RMSE values across the sampled parameter space is shown in **Fig. A1**.

**Fig. A1.** Distribution of root-mean-square error (RMSE) values obtained during the first-stage broad Monte Carlo sampling (n = 5000 samples)

To define the region for the second-stage search, the best 50 valid samples from the broad Monte Carlo stage were used to construct refined bounds: $A$∈ [1.00, 4.23], $\tau_{mrc}$ ∈ [0.25, 0.73] s, and $t_{0,pes}$ ∈ [0.05, 0.46] s. Within these refined bounds, a second Monte Carlo sampling stage was performed using 1000 samples, all of which yielded a finite RMSE. The refined distribution of candidate solutions is shown in **Fig. A2**. The best parameter set obtained from this focused sampling stage yielded an RMSE of 11.4%, with parameter values $A$ = 2.69, $\tau_{mrc}$ = 0.33 s, and $t_{0,pes}$= 0.36 s.

**Fig. A2.** Distribution of root-mean-square error (RMSE) values obtained during the second-stage focused Monte Carlo sampling (n = 1000 samples).

In the third stage, this best focused-sampling solution was used as the center point for a multi-start trust-region Newton conjugate-gradient (trust-ncg) optimization. For each optimization run, the initial parameter vector was randomly drawn from a normal distribution centered on the best focused-sampling solution with a standard deviation of 0.3. A total of 20 optimization runs were performed, all of which converged successfully. The objective function was defined as the sum of squared differences between simulated and experimental mechanical restitution curves, using 61 valid (ESI, PESI) data points. The resulting parameter values and RMSE across all 20 runs are shown in **Fig. A3**.

**Fig. A3.** Converged parameter values and root-mean-square error (RMSE) across all 20 successful multi-start trust-region Newton conjugate-gradient (trust-ncg) optimization runs, initialized from a normal distribution centered on the best focused-sampling solution with a standard deviation of 0.3. Dashed red lines indicate the mean across runs; shaded regions indicate ±1 standard deviation.

The optimization consistently converged to similar solutions across all runs, with means and standard deviations of $A$ = 2.35 ± 0.10, $\tau_{mrc}$ = 0.36 ± 0.01 s, and $t_{0,pes}$ = 0.32 ± 0.01 s, and a mean RMSE of 9.59 ± 0.08%. The final optimal parameter set was selected as the run achieving the lowest RMSE, yielding $A$ = 2.28, $\tau_{mrc}$ = 0.36 s, $t_{0,pes}$= 0.31 s, and $B$ = 1.08 (derived from the steady-state constraint), with a final RMSE of 9.54%. The narrow standard deviations for all three parameters, in particular for $\tau_{mrc}$ and $t_{0,pes}$, confirm that the optimisation reliably converges to the same region of the parameter space regardless of the random starting point, supporting the uniqueness of the identified solution.
